# Sequencing and characterization of the guppy (*Poecilia reticulata*) transcriptome

**DOI:** 10.1186/1471-2164-12-202

**Published:** 2011-04-20

**Authors:** Bonnie A Fraser, Cameron J Weadick, Ilana Janowitz, F Helen Rodd, Kimberly A Hughes

**Affiliations:** 1 Department of Biological Science, Florida State University, Tallahassee, FL, USA; 2 Department of Ecology and Evolution, University of Toronto, Toronto, ON, Canada

## Abstract

**Background:**

Next-generation sequencing is providing researchers with a relatively fast and affordable option for developing genomic resources for organisms that are not among the traditional genetic models. Here we present a *de novo *assembly of the guppy (*Poecilia reticulata*) transcriptome using 454 sequence reads, and we evaluate potential uses of this transcriptome, including detection of sex-specific transcripts and deployment as a reference for gene expression analysis in guppies and a related species. Guppies have been model organisms in ecology, evolutionary biology, and animal behaviour for over 100 years. An annotated transcriptome and other genomic tools will facilitate understanding the genetic and molecular bases of adaptation and variation in a vertebrate species with a uniquely well known natural history.

**Results:**

We generated approximately 336 Mbp of mRNA sequence data from male brain, male body, female brain, and female body. The resulting 1,162,670 reads assembled into 54,921 contigs, creating a reference transcriptome for the guppy with an average read depth of 28×. We annotated nearly 40% of this reference transcriptome by searching protein and gene ontology databases. Using this annotated transcriptome database, we identified candidate genes of interest to the guppy research community, putative single nucleotide polymorphisms (SNPs), and male-specific expressed genes. We also showed that our reference transcriptome can be used for RNA-sequencing-based analysis of differential gene expression. We identified transcripts that, in juveniles, are regulated differently in the presence and absence of an important predator, *Rivulus hartii*, including two genes implicated in stress response. For each sample in the RNA-seq study, >50% of high-quality reads mapped to unique sequences in the reference database with high confidence. In addition, we evaluated the use of the guppy reference transcriptome for gene expression analyses in a congeneric species, the sailfin molly (*Poecilia latipinna*). Over 40% of reads from the sailfin molly sample aligned to the guppy transcriptome.

**Conclusions:**

We show that next-generation sequencing provided a reliable and broad reference transcriptome. This resource allowed us to identify candidate gene variants, SNPs in coding regions, and sex-specific gene expression, and permitted quantitative analysis of differential gene expression.

## Background

Understanding the genetic basis of phenotypic variation is a major challenge for modern biology. Phenotypes result from interactions between genes and environment during development and from interactions between genes and natural selection over evolutionary time. Two barriers to building a complete understanding of phenotypic variation are that (1) single genes rarely act alone in building a phenotype, so genome-wide information is needed, and (2) those organisms for which we have the best ecological knowledge are not generally those for which we have the best genomic knowledge. Sequencing entire genomes of non-model organisms is still out of reach for most researchers but sequencing smaller subsets of the genome, like transcriptomes, provides an attractive alternative. Transcriptomes correspond to the transcribed DNA of an organism and therefore represent functional genomic data. *De novo *assembly and annotation is easier for transcribed genes than for complete genomes because new sequences can be compared to conserved protein sequences and transcribed genes contain fewer repetitive elements.

Recent advances in DNA sequencing technology have reduced the cost and time associated with gathering large amounts of sequence data. For example a single run of 454 GS FLX technology can generate nearly 100 Mbp [1]. Development of transcriptomes in "ecological model species" can provide access to functional and evolutionary analyses previously restricted to genetic model organisms. A well characterized transcriptome can help identify genes underlying phenotypic variation in several ways. Candidate genes that have been identified and characterized in model organisms can be identified in transcriptome databases and tested for signatures of selection in wild populations. Genome-wide scans of selection can identify genes involved in adaptations to specific environments [2]. RNA sequencing (RNA-Seq) can produce short sequences that can be aligned to a reference transcriptome, and used as an assay for genome-wide RNA expression [3]. Creating a reference transcriptome can therefore be an invaluable tool for deciphering the genetic architecture of adaptive traits in species for which complete genome sequence is not available.

Several decades of research on the Trinidadian guppy (*Poecilia reticulata*) have established the species as a model system in evolutionary biology, ecology, and animal behaviour [4,5]. Extensive documentation of parallel evolution along a repeated environmental gradient is one reason that guppies are an important evolutionary model. Waterfalls, characteristic of streams in northern Trinidad, separate guppies into populations that differ in predation and other ecological factors [reviewed in [6]]. Comparative, common garden experiments and transplant studies have revealed parallel evolution of life history traits [see, e.g., [7,8]], male colour patterns [see, e.g., [9,10]] and behaviour [see, e.g., [11]] in response to the ecological differences above and below barrier waterfalls. This research has provided textbook examples of the operation of natural selection [e.g., [12]].

Despite their importance in ecology and evolutionary biology, few genetic or genomic tools have been available for guppies until very recently. These resources currently include approximately 18,000 expressed genes with annotation [13] and a SNP-based genetic map useful for QTL mapping [14-16] and population genetic analyses [17]. Our goal here was to add to these resources by describing a *de novo *assembly of the guppy transcriptome and to test the resulting database for its completeness and its utility for several genome-scale analyses.

To assess the completeness of the transcriptome database, we compared it with those for other fish species and annotated it by searching protein and gene ontology databases. We also identified putative single nucleotide polymorphisms (SNPs) and sex-specific transcripts. Identifying sex-specific or sex-biased genes can shed light on sex determination, sexual dimorphism, and sex-specific selection [18]. In addition, we identified candidate genes of particular interest to the guppy research community. Guppies are important models for studies of mate choice and other social behaviours, sexual selection and sexual dimorphism of colour patterns, and the effects of parasitism on fitness [see [4] and [5] for reviews]. We therefore report transcripts that are candidates for genes implicated in behaviour, colour vision, skin colouration, and parasite resistance.

In addition to sequencing and annotating the transcriptome, we used Illumina short-read sequencing to show that the assembled transcriptome can be a reliable reference for RNA-seq analysis of differential gene expression, both in guppies and in a related species, the sailfin molly (*Poecilia latipinna*). RNA-seq does not depend on the hybridization chemistry of a microarray probe and therefore can detect expression variation over a large dynamic range [3]. Another advantage is that *a priori *knowledge about what genes will be expressed is not necessary, a particular asset in organisms for which a complete genome assembly is lacking. Despite clear benefits, efficient application of this technique to organisms without full genome assemblies has not been demonstrated. To address this issue, we present data on gene expression response in juvenile guppies exposed to a natural predator, *Rivulus hartii*. Anti-predator adaptation has been a major theme in guppy research for many years [reviewed in [5]], and these data contribute to a larger research program intended to elucidate anti-predator behaviour and adaptation.

Finally, we assessed the extent to which the guppy transcriptome could provide a reliable reference for gene expression studies in related species. We mapped RNA-seq data from a congeneric species (the sailfin molly) to the guppy reference transcriptome. Although microarray chips have been used for distantly related taxa with varying success [19] the usefulness of a *de novo *reference transcriptome for studies of related taxa has not yet been determined.

## Results

### Transcriptome Assembly

Guppies used in the sequenced samples were from several natural populations encompassing the three major Trinidadian river drainages; some were wild caught and the others reared in the lab under a range of conditions designed to maximize representation of expressed genes (Additional file 1). Four separate normalized cDNA libraries were created from pooled adult tissue: male brain, male body, female brain, and female body. These libraries were sequenced on four separate plates, one library per plate, by 454 GS FLX technology to produce 366 Mbp of sequence. A total of 1,162,670 reads were assembled into contigs (Table 1, Additional file 2); 171,305 high quality reads were not assembled (singletons) and were excluded from further analysis.

**Table 1:** Run and assembly statistics for 454 sequencing used for the transcriptome assembly.

Total reads (n)	1,665,609
Total bases (bp)	336,869,979
Assembled reads (n)	1,162,670
Bases assembled (bp)	25,534,864
Singletons (n)	171,305
Total contigs (n)	54,987

The assembly produced 54,921 contigs after excluding 66 contigs that were less than 100 bp long and less than 2 reads deep (Table 1). The average length of the remaining contigs was (mean ± standard deviation) 464.81 ± 312.2 bp (range 100-3,571 bp), and the N50 of the assembly was 846 bp. The longest 10% of contigs were 892-3,571 bp (*n *= 5,492). The average number of reads per contig was 28.3 ± 57.6 (range 2-2,110 number of reads). Contigs in the top 10% of number of reads ranged from 72-2,110 number of reads (*n *= 5,508). Number of reads was significantly correlated with length of contig (Pearsons ρ = 0.39; *n *= 54,921; *p *< 0.0001). Short-read files were deposited in the Sequence Read Archive on Genbank (Study Accession ID: SRP005402). Contigs and read files are also available from our website: http://www.bio.fsu.edu/kahughes/Databases.html.

### Comparison to Reference Genomes

To our contig data, we added 16,217 guppy expressed sequence tag (EST) sequences available from Genbank for a total of 71,138 sequences. We compared this full dataset to the medaka (*Oryzias latipes*), three-spined stickleback (*Gasterosteus aculeatus*), and zebrafish (*Danio rerio*) Unigene records [20]. Using blastn similarity searches (both query and reference sequences are nucleotides), we found that 20,859 sequences had matches in the medaka unigene database. These sequences matched 8,344 unique medaka records, covering 37.5% (= 8,344/22,239) of the medaka transcriptome. Of these 4,980 were matched by single guppy sequences and the remainder were matched by multiple guppy sequences; 6,379 reciprocal best-hit matches were identified. Using tblastx similarity searches (both query and reference sequences are translated to amino acid sequences), we found that 24,416 sequences had matches in the medaka Unigene database, corresponding to 9,188 unique medaka records, covering 41.3% (= 9,187/22,239) of the medaka transcriptome. Of these 5,050 matched single guppy reference sequences and the remainder matched multiple sequences.

Similar results emerged when we searched the three-spined stickleback Unigene database. Using blastn we found that 19,654 sequences had matches in the stickleback database. These sequences matched to 7,469 unique stickleback records, covering 39.4% (= 7,469/18,938) of the stickleback transcriptome. Of these 4,450 matched to single guppy reference sequences and with the remainder matching multiple sequences; 5,841 reciprocal best hit matches were found. Using tblastx we found that 23,001 sequences had matches. These sequences matched 7,741 unique stickleback records, covering 40.9% (= 7,741/18,938) of the transcriptome. Of these 4,050 matched single guppy reference sequences, and with the remainder matched multiple sequences.

The zebrafish Unigene records yielded more matches than did the other two databases, but they corresponded to a smaller percentage of the entire zebrafish transcriptome. Using blastn we found that 20,603 sequences had matches in the zebrafish database. These sequences matched to 8,037 unique zebrafish records, covering 15.6% (= 8,037/51,481) of the zebrafish transcriptome. Of these 4,830 zebrafish sequences matched to single guppy reference sequences; 5,525 reciprocal best hit matches were found. Similarly, using tblastx, we found that 27,035 sequences had matches. These sequences matched to 10,532 unique zebrafish records, covering 20.5% (= 10,532/51,481) of the transcriptome. Of these 5,875 matched to single guppy reference sequences, and the remainder were matched by multiple reference sequences.

### Annotation

We annotated our database by first searching the Swiss-Prot [21] and then the NCBI non-redundant (NR) protein [22] databases using blastx. We found that 22,872 (32%) sequences had matches, with 10,008 unique records, in the Swiss-Prot database. An additional 3,569 (5%) sequences had matches in the NR database and matched to 2,791 unique records. In total, 26,445 (37%) sequences were annotated and corresponded to 12,799 unique Swiss-Prot or NR records. If multiple guppy sequences matched the same record in either database, we grouped these sequences into "clusters" so that each cluster represented a unique match. Taxa with the most matches were human (*Homo sapiens*; 2,815 matches, 22%), mouse (*Mus musculus*; 1,802 matches, 14%), and zebrafish (1,563 matches, 12%), where percentages are based on the top hit for each annotated reference sequence. In addition, 737 clusters matched records annotated as hypothetical proteins, 216 as uncharacterized proteins, and 10 as unknown proteins.

**Figure 1:**
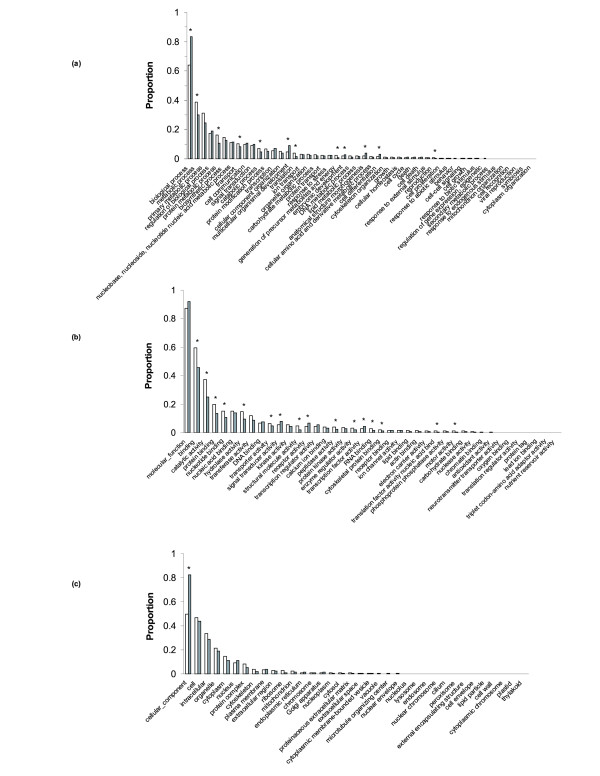
**Gene ontology (GO) ID representations for our guppy transcriptome database (white) and the zebrafish transcriptome (grey)**. Three comparisons are shown: (a) biological processes ontology; (b) molecular function ontology; (c) cellular component ontology. Asterisks denote significant differences between species for each category. Significance was determined via χ^2 ^tests with a *p*-value corrected for multiple tests.

Guppy sequences that had matches in either the Swiss-Prot or NR databases were annotated with Gene Ontology (GO) annotations with the Uniprot database [23]. Of these, 22,029 of 22,773 (83%) were annotated with GO IDs corresponding to 10,442 unique matches in the Uniprot database. These unique matches were then grouped into generic GO terms (GO slims) [24] (Figure 1). We found that 5,201 (49.8%) records were annotated with a cellular component (GO:005575), 9,120 (87.4%) with a molecular function (GO:0003674), and 6,673 (63.9%) with a biological process (GO:008150).

Representation of GO categories in the guppy transcriptome set was similar to that in the zebrafish GO gene association database with a few categories over- or underrepresented in each of the three main GO categories (Figure 1). After correcting for multiple tests, we found that 39 of the 120 comparisons were significantly over or underrepresented in comparison to the zebrafish records. For example, in the biological-processes category, protein metabolic processes (GO:0019538) and catabolic processes (GO:009056) were overexpressed in the guppy data, and multicellular organismal development (GO:0007275) and embryonic development (GO:0009790) were underrepresented.

We next investigated the utility of the guppy transcriptome data set for identifying candidate genes by searching for a subset of genes of particular interest to the guppy research community. Guppies have been used extensively in studies of mate choice, sexual selection, and parasite-mediated selection so we searched the transcriptome for candidate genes involved in visual communication, male ornaments, parasite resistance, and behavioural activation. We found three clusters annotated as nonvisual opsins and eight as visual opsins, eight implicated in pigment synthesis (four in melanin synthesis and four in pteridine synthesis), 14 major histocompatibility complex genes (eight MHC class I, and six MHC class II), four clusters implicated in the regulation of behaviour or in behavioural activation, and two immediate early genes (1 *EGR1*, and 2 *c-fos*) (Table 2).

**Table 2:** Candidate genes with annotations from the Swiss-Prot on NR database (with examples of candidate gene studies).

	Gene description	Accession number	Number in cluster	Database	E-value	Percent coverage
Non-visual opsins[59-61]	Kallikrein-8; AltName: Neuropsin;	O88780.1	1	SP	6e-16	93%
	Kallikrein-8; Short = mK8; AltName: Neuropsin	Q61955.1	1	SP	2e-12	81%
	Melanopsin-like; AltName: Opsin-4-like	Q1JPS6.1	1	SP	8e-43	23%
Visual opsins	Green-sensitive opsin-1; AltName: Green cone	P32311.1	12	SP	6e-87	60%
	Rhodopsin	P79756.1	1	SP	e-172	65%
	Rhodopsin	P79848.1	183	SP	e-64	65%
	Blue-sensitive opsin; AltName: Blue cone	P87365.1	4	SP	e-154	66%
	Green-sensitive opsin; AltName: Green cone	P87366.1	13	SP	9e-108	79%
	Green-sensitive opsin-4; AltName: Green cone	Q9W6A6.2	2	SP	3e-61	32%
	Red-sensitive opsin; AltName: Red cone	P87367.1	6	SP	1e-76	75%
	Putative violet-sensitive opsin; AltName: Violet	P87368.1	12	SP	2e-85	80%
MHC class I [62,63]	Mhc, class IA	CAA90790.1	2	NR	5e-100	20%
	Mhc, class IA	CAA90793.1	1	NR	6e-27	55%
	Mhc, class 1b	CAA90782.1	1	NR	3e-16	31%
	classical MHC class I antigen	ACN49159.1	1	NR	1e-04	9%
	classical MHC class I antigen	ACN49175.1	1	NR	3e-11	26%
	MHC class I related gene	O19477.2	5	SP	4e-8	21%
	MHC class I related gene	Q5RD09.1	1	SP	8e-14	51%
	MHC class I receptor	AAY79253.1	1	NR	5e-15	31%
MHC class II	MHC class II alpha subunit	AAO19852.1	1	NR	1e-8	12%
	MHC class II antigen	AAP20186.1	1	NR	1e-10	17%
	MHC II invariant chain	AAS77256.1	1	NR	5e-22	31%
	MHC class II antigen beta chain	ABX44766.1	1	NR	2e-7	30%
	MHC class II antigen	ACN72667	1	NR	9e-15	41%
	HLA class II histocompatibility antigen, DRB1-8 beta	Q30134.2	2	SP	2e-7	50%
Behaviour Genes [64-67]*Dopamine receptors*	D(4) dopamine receptor; AltName: Dopamine D4	P21917.2	1	SP	2e-22	15%
	D(2)-like dopamine receptor	P53453.1	2	SP	4e-61	28%
*Immediate early genes (and related genes)*	Early growth response protein 1	P26632.2	1	SP	2e-42	24%
	Target of EGR1 protein 1	Q17QN2.1	1	SP	6e-20	37%
	Target of EGR1 protein 1	Q96GM8.1	1	SP	1e-76	38%
	Proto-oncogene protein c-fos	P53450.1	3	SP	8e-30	37%
	c-FosLb protein	CAD56866.1	1	NR	9e-11	14%
Pigment genes [68]*Melanin synthesis*	D-dopachrome decarboxylase-A	Q68FI3.1	1	SP	1e-33	100%
	Melanocyte protein Pmel 17; AltName: Silver	Q98917.3	11	SP	9e-15	14%
	L-dopachrome tautomerase	O93505.1	2	SP	5e-57	29%
	Melanocyte-stimulating hormone receptor	P55167.1	1	SP	2e-36	36%
*Pteridine synthesis*	Dihydropteridine reductase; AltName: HDHPR	P09417.2	2	SP	5e-57	74%
	Pterin-4-alpha-carbinolamine dehydratase	Q91901.3	4	SP	1e-14	52%
	Pterin-4-alpha-carbinolamine dehydratase 2	Q9CZL5.2	1	SP	2e-40	76%
	Putative pterin-4-alpha-carbinolamine dehydratase	Q9TZH6.3	1	SP	5e-41	58%

Presented are the gene description found in the protein database, the accession number, the number of contigs and EST sequences in that annotation cluster, the database (Swiss-Prot: SP, non-redundant: NR), the mean e-value, and mean percent coverage of the reference sequence by the contigs or EST sequences.

### Male-specific Expression

In the 454 assembly, we found 20 contigs that were assembled from male sequence reads only, which were therefore candidates for male-specific expression. Five of these contigs were annotated in the Swiss-Prot or NR database (Table 3). For one, the most significant match was a hypothetical protein and the second most significant match is therefore reported. We chose these five annotated contigs plus one additional contig that had a high depth of coverage (> 100 reads) to test for male specific expression using PCR amplification on whole body homogenate. We confirmed that expression was male-specific for three of these contigs, whereas the remaining three were expressed in both males and females (Table 3, Additional file 3).

**Table 3:** Contigs tested for male specificity by PCR

ID	Gene Description (accession ID)	Number of reads	Length	Number of putative SNPs	Male-specific expression confirmed
contig44905	lipocalin-type prostaglandin D synthase-like protein (second top search) (BAB88224.1)	59	781	3	Yes
contig50719	Islet amyloid polypeptide precursor (ACO09255.1)	63	339	8	No
contig44896	Putative heparin-binding growth factor 1 (Q6PBT8.1)	64	711	2	Yes
contig42251	EF-hand calcium-binding domain-containing protein 2 (Q9CQ46.1)	78	394	2	No
contig50654	Hyaluronidase-2 precursor (AC132917.1)	135	1,465	21	Yes
contig40220	N/A	181	1,179	0	No
					

Presented are the annotations with either the Swiss-Prot or NR databases, the number of reads used to assemble the contig, length of contig, the number of putative SNPs, and whether male-specific expression was confirmed with PCR.

### SNP Discovery

We searched the contigs generated from 454 sequencing for single nucleotide polymorphisms using Mosaik and gigabayes [25]. We considered putative SNPs that had a Bayesian probability of above 0.9 and greater than 4× coverage. We found 11,685 putative SNPs meeting these criteria (Additional file 4). The mean coverage per SNP was 20.94 ± 0.24. A total of 3,956 contigs had at least 1 SNP. The mean number of SNPs per contig was 2.95 ± 0.04. Four contigs had more than 20 SNPs, and 60 contigs had more than 10 SNPs. We report the number of putative SNPs in male-specific and male-biased transcripts in Table 3.

To test our prediction that gene classes thought to be under diversifying natural selection (e.g. MHC genes) would have higher numbers of SNPs, we compared the proportion of contigs without SNPs to the proportion of contigs with SNPs in generic GO slim terms by means of a chi-squared test. Among the 9,164 contigs possessing SNPs that were annotated with uniprot GOIDs, we found no significant difference between the number of contigs with SNPs and the number without in any GO slim term (*p *> 0.05).

### Differential gene expression analysis with RNA-Seq

To investigate the utility of the guppy transcriptome for use in RNA-seq based gene expression analysis, we conducted a small RNA-seq experiment and mapped the resulting reads to a non-redundant version of our transcriptome data. We compared two groups of juvenile fish, one reared in the presence of visual and chemical cues produced by *Rivulus hartii *(which is known to prey on juvenile guppies), and one reared identically but without predator cues. Each treatment group had two biological replicates, and each replicate consisted of mRNA extracted from whole-head homogenate of a mean of two individuals per replicate. We also sequenced one sample of whole-head homogenate taken from two sailfin mollies to determine the utility of the guppy transcriptome data for gene expression analysis in a related species. The Trinidadian guppy and sailfin molly are estimated to have diverged 25 MYA [26]. In total, 124,784,478 high quality reads were obtained from for the guppy samples and 29,754,476 high quality reads was obtained for the molly sample (Table 4). Short Read archive accession numbers is: Study Accession ID: SRP005402.

**Table 4:** RNA-seq results showing the number of reads after the purity filter, the number of reads aligned to our reference database with percent of total number of reads in brackets, and the number of reference sequences the reads mapped to in our database with percentage of total number of sequences in brackets.

	Number of reads (after purity filter)	Number of reads mapped (%)	Number of reference sequences matched (%)
Guppy pred - 1	32,054,094	16,229,906 (51%)	42,501 (73%)
Guppy pred - 2	31,238,411	16,158,014 (51%)	42,256 (72%)
Guppy pred + 1	30,811,092	15,603,608 (51%)	42,072 (72%)
Guppy pred + 2	30,680,881	16,752,768 (55%)	43,099 (74%)
Sailfin molly	29,754,476	12,248,933 (41%)	39,704 (68%)

To generate a non-redundant reference database for the RNA-seq analysis, we performed a self blast search, using an E-value cut off 0.0001, of the entire sequence database (454 contigs and EST data). If sequences were more than 90% identical and overlapped by more than 80% (of the smallest sequence), they were grouped together. The longest sequence of the group (or the first one alphanumerically if they were the same length) was retained in the database, and the redundant sequences were removed. The reduced reference database contained 58,303 sequences (12,901 sequences were removed).

We mapped RNA-seq reads to the reduced database using BWA [27] with default settings. For each guppy sample, a mean of 16,186,074 reads mapped to a unique sequence in the reference database with high confidence, corresponding to 52% of high quality reads. Over all four guppy samples, 48,263 reference sequences (83% of the reduced database) had reads align to them. For the sailfin molly sample, > 40% of reads mapped to the guppy transcriptome, and reads mapped to nearly 40,000 unique reference sequences (Table 4).

To test for differences in counts between treatment groups in the predator-exposure experiment, we used generalized linear models [28] and an empirical Bayesian technique EdgeR [29]. Both approaches model the distribution of count data as negative-binomial or Poisson and account for differences between samples in the total number of reads ("library size"). We chose the negative binomial distribution in both analyses because our experiment included both biological and technical replication, and most genes were over-dispersed relative to the Poisson expectation (data not shown). EdgeR applies an empirical Bayesian method to moderate dispersion estimates across reference sequences by borrowing information across all sequences in the analysis. This moderation improves the reliability of inference in small- to moderate-sized experiments [29].

To determine whether the data contained a signal of differential expression, we first evaluated the distribution of *p-*values obtained from generalized linear models applied to the counts for each transcript, as did Bullard et al. [28]. Additional file 5 shows that this distribution is enriched for small *p *values, indicating that the data contain transcripts that are truly differently expressed in different treatment groups. If expression were not truly different, we would expect the distribution of *p *values to be approximately uniform.

**Figure 2:**
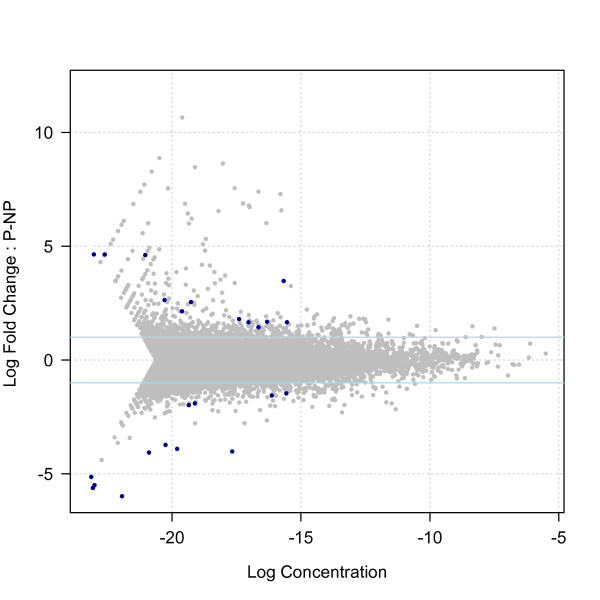
**Differential expression in predator-exposed and non-exposed fish**. The differently expressed genes are in blue, and the others in grey. The x-axis is an estimate of the relative abundance of the transcript (a measure of the average expression level for each sequence across the two groups, A_g_), and the y-axis is a measure of differential expression, M_g_. The solid light-blue horizontal lines show where genes with 2-fold differences in expression would fall, so all the genes with differential expression in this analysis show > 2 fold differences between treatments. Reference sequences with very low or very high values of M_g _have their fold-change values compressed to fit within the [-10, +10] interval. The compressed values usually represent sequences with zero counts in one treatment group.

We used EdgeR to identify reference sequences showing the strongest evidence for differential expression. We found that the number of reference sequences classified as differently expressed depended strongly on the degree of moderation applied to the dispersion estimates. With strong moderation (relative weight of the common versus sequence-specific dispersion estimates = 10:1), 388 reference sequences were differently expressed at *p *< 0.01; 92 of these remained significant after correction for multiple testing by the method of Benjamini and Hochberg [30] to control the false discovery rate (FDR). With weak moderation (relative weight = 1:1), we found 300 reference sequences differently expressed at *p *< 0.01; 24 of these had FDR < 0.05 after correction for multiple tests (Figure 2).

Of the 24 reference sequences identified as differentially expressed with the more conservative model, 12 had higher counts in the predator-exposed treatment (mean log_2 _of fold change = 2.4 ± 1.0), and 12 had higher counts in the non-predator-exposed treatment (mean log_2 _of fold change = 3.2 ± 1.5). Annotations and counts for these 24 genes are given in Table 5 and, Figure 2.

**Table 5:** List of sequences that were differently expressed in guppies exposed to predators and guppies that were not

Sequence name	Log FC	*P*-value	FDR	Counts pred - 1	Counts pred - 2	Counts pred + 1	Counts pred + 2	Annotation (accession #)
contig36557	-30.67	2e-08	0.0004	16	32	0	0	
ES381343	-4.02	3e-08	0.0004	175	443	22	16	hepcidin-like precursor (AAS66305.1)
contig46890	-30.54	1.1e-07	0.0009	14	30	0	0	
contig41409	4.61	1.1e-07	0.0009	3	0	25	49	
contig34882	-5.98	1.3e-07	0.0009	19	44	0	1	
contig40497	1.66	1.9e-07	0.0010	184	193	524	671	Cerebellin-2 (Q8BGU2.1)
contig34536	-3.73	2.4e-07	0.0011	33	60	5	2	hepcidin-like precursor (AAS66305.1)
contig30446	-3.9	5.4e-07	0.0022	41	94	6	3	LINE-1 reverse transcriptase homolog (P08548.1)
ES374452	2.54	6.7e-07	0.0024	12	9	69	53	Fibrocystin-L (Q86WI1.1)
ES383031	3.47	1.7e-06	0.0054	94	90	493	1588	Nattectin Precursor (Q66S03.1)
contig06616	1.8	2.3e-06	0.0068	47	52	146	200	Cerebellin-1; (P63182.2)
ES376890	-1.47	3.7e-06	0.0098	493	601	196	200	Fibronectin (P07589.4)
contig32843	2.63	4.3e-06	0.0106	5	5	31	31	
contig46202	29.67	4.9e-06	0.0111	0	0	10	14	
ES371621	-1.56	5.2e-06	0.0111	345	419	135	124	Proactivator polypeptide (P07602.2)
contig18751	1.66	8.4e-06	0.0168	70	64	176	249	
contig36338	-30.17	1.6e-05	0.0294	8	26	0	0	
contig38849	-1.91	2.1e-05	0.0352	53	56	16	13	Complement C1q tumor necrosis factor-related protein (P0C862.1)
ES371258	1.68	2.1e-05	0.0352	132	87	312	392	
contig40097	2.14	2.2e-05	0.0352	10	9	36	48	Granzyme A (P11032.2)
contig13556	1.44	3.2e-05	0.0494	97	92	216	299	
contig37489	-1.98	3.5e-05	0.0494	47	48	14	10	
contig33977	4.64	3.6e-05	0.0494	1	0	10	15	
ES383122	-4.06	3.7e-05	0.0494	17	50	3	1	60S ribosomal protein L11 (Q5RC11)

Shown is the log fold change (Log FC) of the number of counts, *p*-value, corrected *p*-value (FDR), counts for samples not exposed to predators (pred - 1 and pred - 2) and exposed to predators (pred + 1 and pred + 2). Annotation is from either the Swiss-prot or the NR databases with their accession number in brackets.

Our ability to detect differential gene expression was not restricted to high-abundance transcripts. In fact, Figure 2 shows that differential expression was more likely to be detected at low to moderate abundance. We also found little bias in favour of detecting differential expression for longer genes or reference sequences, as has been reported in some studies [31]. The length of the reference sequence was very slightly but significantly correlated with the number of counts (Spearman's ρ = 0.01, *p *= 0.03, *n *= 32,217).

Using the reduced reference database should have decreased the likelihood that a single read would map to multiple reference sequences. Nevertheless, we tested for bias that could result from eliminating any read with multiple hits by including all mapped reads in a reanalysis of the RNA-seq data. The only substantive change in the results was that one reference sequence (un-annotated contig34708) that did not meet the FDR < 0.05 cut-off in the original analysis did meet that cut-off in the re-analysis.

## Discussion

Using next generation sequencing technologies, we were able to sequence and annotate a reference transcriptome for the guppy. Approximately, 336 Mbp were sequenced to create the reference transcriptome and then an additional 9 Gbp were sequenced in the gene expression analysis. Our results represent the most extensive sequencing resource published for the guppy and indeed one of the largest sequencing projects for a non-model species (i.e. for a species for which no genomic resource is available). By means of next generation sequence technologies, *de novo *transcriptome assemblies have been created for several such species in a variety of taxa: plants (*Eucalyptus grandis *[32]; *Castanea dentata*, *C. mollissima *[33]; *Pinus contorta *[34]; *Panax quinquefolius *[35]), insects (*Melitaea cinxia *[36]; *Sarcophaga crassipalpis *[37]; *Erynnis propertius*, *Papilio zelicaon *[38]), coral (*Acropora millepora *[39]), a marine gastropod (*Littorina saxatilis *[40]), fishes (*Zoarces viviparous *[41]; *Amphilophus zaliosus, A. astorquii *[42]; *Coregonus *sp. [43]), and 10 species of birds [44].

Reference transcriptomes can be developed by assembly of the short sequence reads typical of next-generation sequencing technology into longer contiguous sequences more representative of a complete transcript. The average length of contigs from our study (464 bp) is comparable to those of other studies using similar technologies (mean = 366 bp, range 197-714 bp [32-44]). We obtained a greater depth of coverage per contig than did these other studies. The average number of reads per contig was 28, higher than that of other reference transcriptomes previously reported (mean = 8, range = 3-13 [32-44]). We were able to confirm the presence of 11 (out of 11) contigs by PCR. This validation and high degree of coverage suggest that our contigs are representative of real transcripts and not due to assembly error.

Our reference transcriptome represents an extensive sampling of the guppy transcriptome. By comparing it to those of species with well characterized genomes, we estimated that we have recovered roughly 40% of the entire transcriptome, corresponding to 8,000 unique unigenes. This number is probably an underestimate, however, because many guppy transcripts would probably not align to transcripts of species as divergent as the medaka, three-spined stickleback, or zebrafish (estimated to have diverged from the guppy 70 MYA, 100 MYA and 175 MYA, respectively; [45]). We were able to match 37% of our database with functional annotations in the Swiss-Prot, NR protein, and GO databases, a figure comparable to those from other studies using similar approaches (Swiss-prot and NR = average 33%, range 12 - 73%, GO database = average 20%, range 18 - 33% [32-44]).

While there are some differences between our reference database for the guppy and the available database for the zebrafish in GO annotations, concordance in the overall distributions suggests that our library sampled widely across categories and provides a good representation of the transcriptome. Under-representation of a few GO categories (multicellular organismal development, anatomical structure morphogenesis, and embryonic development) is probably due to the choice of tissues in the two different sources of sequence data that we used. The 454 data used to assemble the transcriptome were derived from adult tissue, whereas the ESTs downloaded from Genbank represent many different developmental stages. Under-representation of genes involved in early development might be the result of the relatively larger amount of sequence derived from adults. Enriching the reference transcriptome with sequence derived from ovaries and developing embryos (removed from our original samples to ensure sex-specific expression) could produce a more complete reference transcriptome

Guppies have been the focus of evolutionary, ecological and behavioural research for over a century, and much of the motivation for developing a transcriptome resource was to provide tools to investigate genetic and genomic bases of adaptive variation. We were able to identify a large number of genes in categories of particular interest to evolutionary and behavioural ecologists, supporting the utility of the transcriptome assembly in these fields. We used a relatively liberal E-value cut-off, however, and, in some cases, the percent coverage of the subject sequence by the candidate gene sequence was relatively low, suggesting caution when these sequences are used in candidate gene studies. Coding-region SNPs are useful markers for mapping studies and for genome wide screens for signatures of selection, and we were able to identify putative SNP markers in nearly 4,000 unique contigs representing a broad sampling of functional categories.

Use of sex-specific libraries for 454 sequencing was moderately successful in identifying genes with sex-specific expression. We identified 20 candidate male-specific transcripts that were assembled from reads originating in male libraries; only 50% of transcripts tested were confirmed to be male-specific by a PCR assay. A similar approach was taken by Hale and colleagues [46], who found that all 33 contigs unique to either sex's transcriptome assembly in sturgeon (*Acipenser fulvescens*), were expressed in both sexes when tested by PCR.

In contrast, analysis of differential gene expression by mapping of short-read sequences to the reference transcriptome appears to be a robust strategy. On average, 52% of reads mapped to the reference, and this coverage was sufficient to reveal differential expression for transcripts with as few as 24 mapped reads. Wolf and colleagues [47], studying carrion crows (*Corvus corone*), found that 69% of their short-sequence reads hit a zebra finch or chicken reference genome. That study did not report using a mapping quality threshold, however, so the percentage of high-confidence mapped reads could be comparable to our own.

We studied gene expression differences in two treatment groups, one in which juvenile guppies were exposed to predators and one in which they were not. At least two of the differently expressed reference sequences are good candidates for response to the predator exposure treatments: *cerebellin-1 *and *cerebellin-2*, which were both expressed at higher levels in samples from predator exposed fish. *Cerebellin-1 *has been implicated in stress response: it stimulates the production of norepinephrine and increases andrenocortical secretion in rats [48]. *Cerebellin-2 *also has been implicated in regulation of neuronal processes [49]. These results are encouraging in that we were able to obtain significant differences in transcript counts over a broad dynamic range even though the experiment was small. Results of this analysis were sensitive to parameters chosen for the empirical Bayesian estimates of dispersion and somewhat sensitive to exclusion of reads that mapped to multiple reference sequences. This sensitivity suggests that care should be taken in the choice of mapping and analysis parameters, especially in small experiments.

The RNA-Seq data also provided additional confirmation of the quality of the transcriptome assembly. More than 70% of the transcriptome reference was matched by >20 reads in the RNA-Seq data. Because only a single age class of fish was used in this experiment, and only a small subset of tissues were sampled, this result suggests both that a large fraction of the assembled contigs in the data set are accurate, and that the RNA-seq process recovered a high fraction of expressed genes. In addition, the guppy transcriptome appears to be useful for RNA-Seq experiments in related species. Approximately 40% of reads from a sailfin molly sample mapped to the guppy data set.

## Conclusions

Like many organisms that are of great interest in ecological, evolutionary, and behavioural research, guppies and their close relatives lack complete genome sequences and most other genetic tools and resources. Here we show that next-generation sequencing provided a reliable, broad reference transcriptome that we have assembled and annotated. This resource allowed us to identify candidate genes, putative SNPs, sex-specific gene expression, and differential gene expression at a genomic scale. The reference transcriptome also proved useful for transcript mapping in a related species, the sailfin molly.

## Methods

### Transcriptome Sequencing

Samples were taken from one male and one female guppy from each of seven different localities, for a total of 14 fish (Additional file 1). These localities represented a variety of predation regimes, laboratory environments, and exposure to novel objects to ensure that a variety of genes were expressed (Additional file 1). Fish were not fed on the day of dissection. Each fish was netted directly into an ice water bath until no movement was detected (<30 seconds), and was then decapitated. Brains were separated from all other tissues. Ova and embryos were removed from female samples and discarded. Average dissection time was 3.8 min. Samples were stored in RNA-later (Qiagen).

RNA was extracted with the RNA-easy lipid mini kit (Qiagen) and then pooled into 4 samples: male brain, male body, female brain, and female body. cDNA libraries were constructed for each sample by the SMART cDNA amplification technique [50] with some modifications. The first-strand cDNA was generated by with the 5'Smart Oligo, 5'-AAGCAGTGGTAACAACGCATCCGACGCGGG-3' and 3'Oligo dT SmartIIA, 5'AAGCAGTGGTAACAACGCATCCGACTTTTTTTTTTTTTTTTTTTTTT-3'. The cDNA was then normalized with the TRIMMER cDNA Normalization kit (Innovative Biotechnology Company, Moscow, Russia), which uses duplex-specific nuclease (DSN) treatment [51]. The cDNA was amplified with the SmartIIA 5'-AAGCAGTGGTAACAACGCATCCGAC-3' primer. The products of the first run of LD-PCR were purified with the QIAquick PCR Purification Kit. The normalized cDNA was used as a template for the second run of LD-PCR. The SMART II was used as the primer for LD-PCR. The products were purified with the QIAquick PCR Purification Kit. Normalized cDNA was sequenced by the 454 GS FLX system following [1]. Reads were filtered and trimmed of adaptors, primers, and poly-A and poly-T tails; underwent quality trimming; and then were assembled by Newbler with default parameters (ver 2.0, Roche). Average read length was 212.2 before trimming and 201.0 after trimming.

Lab-born fish were reared at the University of Toronto and wild-caught fish were collected in Trinidad, all were dissected at the University of Toronto. RNA was extracted at Florida State University. Library preparation, normalization, and sequencing were conducted at the Genome Center at Washington University in St. Louis.

### Annotation

To the assembled 454 data, we added guppy EST data available from Genbank [ES370951-ES387146] (downloaded on 24 November 2009). We then compared the complete database (contigs and EST data) to NCBI Unigene records for the medaka, three-spined stickleback and zebrafish [[20], downloaded on 10 January 2010]. We conducted similarity searches with tblastx (translating both query and reference database) and blastn (both query and reference database remain nucleotides) with an E-value threshold of 0.001.

To annotate the complete dataset, we searched the curated protein databases Swiss-Prot and NCBI non-redundant (NR). We first matched our sequences to the Swiss-Prot database [21] (blastx, critical E-value = 0.001, downloaded 12 August 2009). The remaining unannotated sequences were matched to the NR database [22] (blastx, critical E-value = 0.001, downloaded 12 August 2009). The protein databases were searched in this order because the NR database is larger than the Swiss-Prot database but contains fewer records with informative functional annotation. We then clustered those of our contigs and ESTs that matched to the same record in either database. We searched clusters for candidate genes using simple text searches based on gene names or synonyms.

Sequences that were matched in the Swiss-Prot or NR database were annotated with Gene Ontology (GO) IDs with the Uniprot-trEMBL protein sequence database [23] (blastx, critical E-value = 0.001, downloaded 20 November 2009). The Uniprot-trEMBL database was annotated with GO terms using the Uniprot annotation file [52] (downloaded 20 November 2009). GO IDs are organized hierarchically; each GO ID is defined by its parent-child relationship for three non-overlapping ontologies (biological process, molecular function, and cellular component). GO IDs can therefore be categorized on the basis of a smaller set of high-level GO terms called a "slim". We used the generic slim (the Gene Ontology ver. 1.0) and the perl script map2slim to categorize the GO IDs for each unique Uniprot record that matched our sequences [[53], downloaded 15 January 2010, [54] downloaded 12 December 2009, and [55], downloaded 15 January 2010]. Additional scripts were modified from Meyer et al. [39] for annotation and clustering.

To estimate the over- or under-representation of records in each generic slim term, we compared the number of unique Uniprot records in each slim term in our guppy database to the number of records in each term found in the zebrafish GO association database [56]. Differences between guppy and zebrafish in the proportion of transcripts falling into GO slim categories were subjected to a χ^2 ^test.

### Male specific expression

To search for sex-specific expression we looked for contigs that were constructed exclusively from reads derived from male samples, with a minimum depth of 50 reads. We then developed conservative primers to test for sex-specific expression (Additional file 6). All candidate male-specific genes were validated by PCR amplification of three independent biological replicates of male and female homogenate samples. Each replicate included tissue pooled from three different individuals. Fish were euthanized and dissected as described above. RNA was extracted with an RNeasy mini kit (Qiagen) according to the manufacturer's instructions, including an on-column DNase treatment. A second DNase treatment was performed with a DNase digestion of RNA before RNA cleanup (Qiagen). Samples for each replicate were then pooled. First-strand cDNA was synthesized from 2 μg of total RNA with SuperScript III First-Strand Synthesis System for RT-PCR (Invitrogen) according to the manufacturer's instructions. End-point RT-PCR reactions contained 1 μl cDNA with 20 μl Platinum PCR SuperMix High Fidelity (Invitrogen) and 1 μl primer (forward and reverse reconstituted and mixed; 10 μM each). PCR cycling conditions were: initial denaturation at 95°C for 1 minute, followed by 28 cycles of 95°C for 30 seconds, 30 seconds at the appropriate annealing temperature (Additional file 6), and a 72°C extension for 30 seconds. A final extension at 72°C for 7 minutes was carried out. Each PCR was run with three controls: male sample without reverse transcriptase, female sample without reverse transcriptase, and a no-template control.

### SNP discovery

Putative SNPs were identified with Mosaik and Gigabayes [25]. 454 reads were first realigned to their contigs with Mosaik because the Newbler assembler inserts an indel in the ace files where base-pair mismatches occur. We investigated putative SNPs that had a Bayesian probability of over 0.9 and greater than 4 fold coverage. These parameters were chosen because we found that setting lower coverage limits or probabilities led to a greatly inflated number of putative SNPs. We then compared the proportion of contigs with SNPs to the proportion without, annotated within generic GO slim terms using χ^2 ^tests.

### Differential gene expression using Illumina short reads

We sequenced mRNA from four guppy samples and one molly sample. Guppy samples consisted of two independent biological replicates from each of two treatment groups: (1) juvenile fish reared in the presence of visual and chemical cues from *Rivulus hartii *(a natural predator of juvenile and mid-sized guppies); and (2) juveniles reared identically, but without exposure to predator cues. Samples consisted of two individuals in non-exposed predator sample 1, one individual in non-exposed predator sample 2, two individuals in predator exposed sample 1, and three individuals in predator exposed sample 2. All fish were descendents of wild fish collected from a tributary of the Paria river in northern Trinidad. After sacrifice, whole heads were dissected, and RNA was extracted with the RNeasy lipid mini kit (Qiagen). The molly sample consisted of head tissue pooled from two different individuals that were collected from Mashes Sands, Florida. For all samples, cDNA was synthesized with the mRNA-seq Sample Prep kit (Illumina), and each of the five samples was run on a separate lane of an Illumina GAII DNA sequencer to generate 36 bp paired-end reads. cDNA synthesis, library preparation and sequencing were conducted by Expression Analysis in Durham, North Carolina. These reads were filtered for purity by removal of any read that contained two low-quality bases in the first 25 bp.

To provide a reference transcriptome for the RNA-seq data, we constructed a "reduced" reference database by performing a self blast search with an E-value cut off 0.0001 of the entire sequence database (454 contigs and EST data). If sequences were more than 90% identical and overlapped more than 80% (of the smallest sequence), they were clustered together. The longest sequence of the cluster (or the first one alphanumerically if they were the same length) was kept in the database, and the redundant sequences were removed.

We mapped the filtered Illumina reads to the reduced database using BWA [57] as implemented in the Galaxy Project [58]. We used default parameters for the mapping and then filtered the mapped reads by mapping quality score [57]. Only reads that mapped uniquely to a single reference sequence and had mapping quality ≥ 37 were included in subsequent analyses. Mapping quality measures the probability that the alignment found is wrong, measured on a Phred scale. Mapq of greater than 37 implies that the alignment is wrong with a probability of < 10^-3.7^. Each end of the paired-end reads for a sample was mapped separately. If both ends of a paired read mapped uniquely to the same reference sequence, the count for that sequence was increased by 1. The reference sequence count was also increased by 1 when only one end of a paired-end read mapped uniquely to the sequence.

To assess the overall signal for differential expression within the set of data, we first filtered reference sequences so that those with very low counts (< 20) in both treatment groups were excluded. For the remaining 32,488 reference sequences, we fit a separate generalized linear model to each sequence, with count as the dependent variable and treatment group as the independent variable. We used the offset parameter in the R function *glm *(R version 2.11.0) to normalize the counts for differences among samples in the total library size, as did Bullard et al. [28].

To identify sequences with the strongest evidence for differential expression, we used the Bioconductor package EdgeR [29], which applies a separate exact test to each reference sequence, but moderates the sequence-specific dispersion (a measure of biological and technical variability). For small experiments such as the one we conducted, the estimate of variability for a single test is based on only a few measurements and is therefore not very reliable. Robinson et al. [29] applied an empirical Bayesian approach to adjust (moderate) the dispersion used in testing each sequence by computing a "common dispersion" estimated from all 32,488 sequences tested. The degree of moderation is adjustable. We first analyzed our data with moderately strong moderation, in which the common dispersion estimate was given 10 times the weight of the sequence-specific dispersion. We then compared that analysis to one with weak moderation, in which the common and sequence-specific dispersion estimates were given equal weight. In EdgeR, the log_2 _fold change for a given sequence is calculated as a weighted trimmed mean of the log expression ration: , where *Y*_*gk *_is the count for sequence *g *in sample *k*, and *N*_*k *_is the total number of reads over all sequences for sample *k*. The absolute expression level for a sequence is calculated as. *A*_*g *_values for sequences with zero count in one of the two groups are estimated by addition of a small rational number to sequence counts in each sample.

This research adhered to the requirements of the Canadian Council of Animal Care for the use of Animals in Research, which follows international guidelines, the legal requirements of Canada and institutional guidelines at the University of Toronto. Protocol numbers were 20006937, 20008230, and 20007873.

## Authors' contributions

BAF conducted the bioinformatics analyses, prepared RNA for the RNA-seq analysis, collected samples, and drafted the manuscript. CJW collected the samples and participated in the bioinformatics. IJ collected and analyzed the samples for the male-specific expression analysis. FHR collected the samples, conceived the study and design, and supervised the work. KAH conceived the study and design, analyzed the RNA-seq data and supervised the work. All authors read and provided advice on the manuscript.

## Supplementary Material

Additional file 1 **Description of sample sources**. Click here for file

Additional file 2 Run statistics for 454 data separately for each sample. Click here for file

Additional file 3 **Male-specific expression tested by PCR**. Each contig tested is represented by an example of the PCR results. The lanes correspond to (1) male cDNA, (2) female cDNA, (3) control male sample without reverse transcriptase, (4) control female sample without reverse transcriptase, and (5) control with no template. Click here for file

Additional file 4 **Summary of the putative single nucleotide polymorphism (SNPs) detected**. Shown is the number of SNPs that were transitions and transversions. Click here for file

Additional file 5 **Distribution of *p*-values from transcript-specific generalized linear models testing**. Click here for file

Additional file 6 **Male specific primer details**. Click here for file
