# Supplementary material for: Sequencing and characterization of the guppy (Poecilia reticulata) transcriptome
Source: BMC Genomics. 2011 Apr 20;12:202. doi: 10.1186/1471-2164-12-202 (PMC3113783; doi:10.1186/1471-2164-12-202)
Supplement: Additional file 1 — Description of sample sources. [file 1471-2164-12-202-S1.DOCX]

Additional file 1: Description of sample sources. One male and one female were taken from each source

| Population | Drainage | Wild/Lab | Predation | Number in stock tank | Novel treatment |
| --- | --- | --- | --- | --- | --- |
| Marianne River | North slope | Wild | High | n/a | No |
| Marianne tributary | North slope | Lab | Low | 5 | No |
| Paria tributary | North slope | Lab | Low | 50 | No |
| Quare River | Oropouche | Lab | High | 4 | No |
| Quare tributary (Q6) | Oropouche | Lab | Low | 15 | Yes |
| Aripo River upstream | Caroni | Lab | Low | 5 | No |
| Aripo River downstream | Caroni | Lab | High | 30 | Yes |
