# Supplementary material for: Sequencing and characterization of the guppy (Poecilia reticulata) transcriptome
Source: BMC Genomics. 2011 Apr 20;12:202. doi: 10.1186/1471-2164-12-202 (PMC3113783; doi:10.1186/1471-2164-12-202)
Supplement: Additional file 2 — Run statistics for 454 data separately for each sample. [file 1471-2164-12-202-S2.DOCX]

Additional file 2: Run statistics for 454 data separately for each sample.

| Sample | Number of reads | Number of  bases | Number of assembled reads | Number of assembled bases |
| --- | --- | --- | --- | --- |
| Male brain | 205,929 | 41,812,125 | 176,838 (85.87%) | 34,236,583 (81.88%) |
| Male brain | 202,652 | 40,594,698 | 172,446 (85.09%) | 33,003,358, (81.30%) |
| Female brain | 209,895 | 42,557,053 | 179,342 (85.44%) | 34,755,712 (81.67%) |
| Female brain | 212,221 | 43,127,379 | 180,623 (85.11%) | 35,076,713 (81.33%) |
| Male body | 239,255 | 47,450,827 | 210,072 (87.80%) | 39,999,450 (84.30%) |
| Male body | 211,442 | 41,474,920 | 185,251 (87.61%) | 34,978,795 (84.34%) |
| Female body | 238,762 | 48,898,499 | 216,413 (90.64%) | 41,520,409 (84.91%) |
| Female body | 145,453 | 30,954,478 | 133,769 (91.97%) | 26,365,330 (85.17%) |
| Total | 1,665,609 | 336,869,979 | 1,454,754 (87.34%) | 279,936,350 (83.10%) |
