# Supplementary material for: Sequencing and characterization of the guppy (Poecilia reticulata) transcriptome
Source: BMC Genomics. 2011 Apr 20;12:202. doi: 10.1186/1471-2164-12-202 (PMC3113783; doi:10.1186/1471-2164-12-202)

Additional file 3: Male-specific expression tested by PCR. Each contig tested is represented by an example of the PCR results. The lanes correspond to (1) male cDNA, (2) female cDNA, (3) control male sample without reverse transcriptase, (4) control female sample without reverse transcriptase, and (5) control with no template.

contig44905- male specific

contig50719- not male specific

contig44896- male specific

contig42251- not male specific

contig50654- male specific

contig40220- not male specific


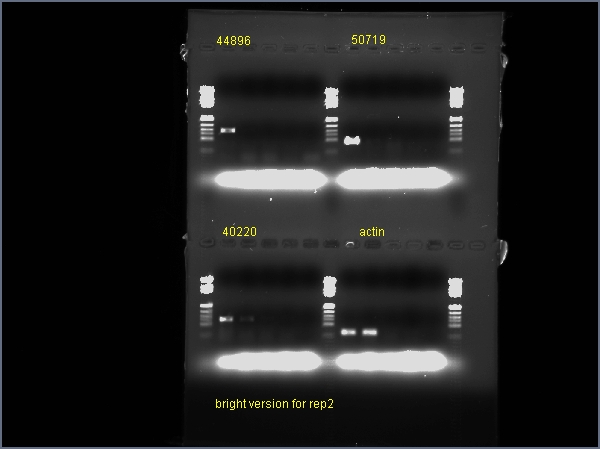

Supplement: Additional file 3 — Male-specific expression tested by PCR. Each contig tested is represented by an example of the PCR results. The lanes correspond to (1) male cDNA, (2) female cDNA, (3) control male sample without reverse transcriptase, (4) control female sample without reverse transcriptase, and (5) control with no template. [file 1471-2164-12-202-S3.DOCX]
