# Supplementary material for: Sequencing and characterization of the guppy (Poecilia reticulata) transcriptome
Source: BMC Genomics. 2011 Apr 20;12:202. doi: 10.1186/1471-2164-12-202 (PMC3113783; doi:10.1186/1471-2164-12-202)
Supplement: Additional file 4 — Summary of the putative single nucleotide polymorphism (SNPs) detected. Shown is the number of SNPs that were transitions and transversions. [file 1471-2164-12-202-S4.DOCX]

Additional file 4: Summary of the putative single nucleotide polymorphism (SNPs) detected. Shown is the number of SNPs that were transitions and transversions.

| Base substitution | *N* |
| --- | --- |
| Transitions |  |
| A-G | 3,683 |
| C-T | 3,788 |
| Transversions |  |
| A-T | 1,129 |
| A-C | 1,100 |
| C-G | 848 |
| T-G | 1,137 |
| Total | 11,685 |
