# Supplementary material for: Sequencing and characterization of the guppy (Poecilia reticulata) transcriptome
Source: BMC Genomics. 2011 Apr 20;12:202. doi: 10.1186/1471-2164-12-202 (PMC3113783; doi:10.1186/1471-2164-12-202)
Supplement: Additional file 5 — Distribution of p-values from transcript-specific generalized linear models testing. [file 1471-2164-12-202-S5.DOCX]

Additional file 5: Distribution of *p*-values from transcript-specific generalized linear models testing the null hypothesis that transcript counts are equal for fish in the two treatment groups: predator exposed and unexposed juvenile fish.
