# Supplementary material for: Sequencing and characterization of the guppy (Poecilia reticulata) transcriptome
Source: BMC Genomics. 2011 Apr 20;12:202. doi: 10.1186/1471-2164-12-202 (PMC3113783; doi:10.1186/1471-2164-12-202)
Supplement: Additional file 6 — Male specific primer details. [file 1471-2164-12-202-S6.DOC]

Additional file 6: Male specific primer details.

| Contig ID | Forward sequence | Reverse sequence | Annealing temperature |
| --- | --- | --- | --- |
| 42251 | 5’-GAAAGGTTTCCTCGAACCCG-3’ | 5’-ACTCTTTCTACTTGGGAGCGTC-3’ | 54°C |
| 50654 | 5’-GGAGTGAAGGTAGCAGGAAC-3’ | 5’-CGTCCTTAGAGGGTGTTGACAC-3’ | 56°C |
| 44905 | 5’-CCCATGACATTGGCACTCAG-3’ | 5’-AACAGTAGAGTCGTTGCAAAGC-3’ | 56°C |
| 44896 | 5’-GGAGAGACGCACGAGAGTCAAG-3’ | 5’-CTTGCCTCGGCAGGAATAAAAC-3’ | 58°C |
| 50719 | 5’-GAGGACGAAACGTTACACCCAG-3’ | 5’-GTGAGCCCGTGAGACCTTCC-3’ | 58°C |
| 40220 | 5’-ATGTGCTGTCTGAAGGTTTGGG-3’ | 5’-TTGCAGTTTGGCACTTTACCAC-3’ | 58°C |
